# The multidimensional mechanisms of long noncoding RNA function

**DOI:** 10.1186/s13059-017-1348-2

**Published:** 2017-10-31

**Authors:** Francesco P. Marchese, Ivan Raimondi, Maite Huarte

**Affiliations:** 1 University of Navarra, Center for Applied Medical Research (CIMA), Pamplona, 31008 Spain; 2 Institute of Health Research of Navarra (IdiSNA), Pamplona, 31008 Spain

## Abstract

A major shift in our understanding of genome regulation has emerged recently. It is now apparent that the majority of cellular transcripts do not code for proteins, and many of them are long noncoding RNAs (lncRNAs). Increasingly, studies suggest that lncRNAs regulate gene expression through diverse mechanisms. We review emerging mechanistic views of lncRNAs in gene regulation in the cell nucleus. We discuss the functional interactions that lncRNAs establish with other molecules as well as the relationship between lncRNA transcription and function. While some of these mechanisms are specific to lncRNAs, others might be shared with other types of genes.

## Introduction

The appreciation of the complexity of the human transcriptome has revolutionized our perception of the regulatory potential of RNA. The efforts to generate a comprehensive atlas of the transcripts expressed in cells have revealed an extremely large collection of lncRNAs [1, 2]. The lncRNAs are broadly defined as noncoding RNA molecules longer than 200 nucleotides. Most of them are transcribed by RNA polymerase II, thus sharing similarities with messenger RNAs (mRNAs)—including a 5′ 7-methylguanosine cap and a 3′ poly(A) tail—however, they lack coding capacity. To date, the ENCODE project (GENCODE v26) has conservatively annotated in humans close to 16,000 lncRNA genes that give rise to more than 28,000 distinct transcripts. Moreover, protein-coding genes too can produce transcript variants that lack coding capacity, adding to the vast catalogue of long noncoding transcripts present in the cells.

**Fig. 1:**
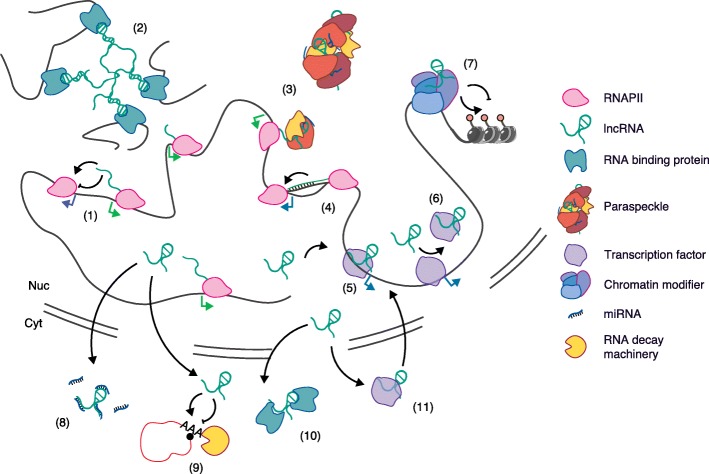
The multiple dimensions of long noncoding RNA (lncRNA) function. lncRNAs can regulate gene expression by different mechanisms, some of which are illustrated here. These modes of action include lncRNA transcription-dependent activation or repression of neighbour genes (*1*), lncRNA-mediated inter-chromosomal interactions (*2*), formation of nuclear structures (i.e. paraspeckles) (*3*) or R-loops (*4*), lncRNAs as guide (*5*) or decoy (*6*) of transcription factors or as a scaffold for chromatin modifying complexes (*7*), lncRNAs acting as sponges of miRNAs (*8*), regulating post-transcriptional mRNA decay (*9*), regulating the cellular localization of RNA-binding proteins (RBPs) (*10*) or DNA-binding proteins (DBPs) (*11*)

Despite not being translated into proteins, lncRNAs are functional molecules. Indeed, since the early studies that demonstrated the central role of *Xist* in the process of X-chromosome inactivation [3, 4], a growing body of evidence has described a myriad of functions for lncRNAs in many cellular processes, such as gene imprinting [5], differentiation and development [6], antiviral response [7], and vernalization in plants [8]. Among the variety of mechanisms reported (Fig. 1; Table 1), many lncRNAs have been shown to interact with chromatin-modifying complexes, to be involved in the conformation of nuclear domains, or in the activity of transcriptional enhancers [9–12]; others have been shown to interfere with the transcriptional machinery or maintain the structure of nuclear speckles [13–15]. Furthermore, some lncRNAs act post-transcriptionally as regulators of splicing, mRNA decay, protein translation, protein stability, or as molecular decoys for microRNAs (reviewed in [16, 17]). What has emerged from all these studies is that lncRNAs are highly heterogeneous and have a substantial functional versatility that relies on their ability as long RNA molecules to conform to different structures and molecular interactions. Moreover, the deregulation of lncRNAs has been related to different human diseases, including cancer and cardiovascular and neurodegenerative diseases [18, 19].

**Table 1:** Long-noncoding-RNA-mediated nuclear mechanisms

Long noncoding RNA	Function	Reference
lncRNAs interacting with chromatin complexes		
*Fendrr*	Differentiation of tissues derived from lateral mesoderm	[34]
*HOTTIP*	Homeotic gene activation at the *HOXA* locus	[35]
*DBE-T*	Epigenetic regulation of the *FSHD* gene	[36]
*Kcnq1ot1*	Lineage-specific imprinting at the *Kcnq1* locus	[37]
*pRNA*	CpG methylation of rRNA genes	[38]
*Morrbid*	Survival control of myeloid cells	[40]
*Linc-Pint*	Epigenetic regulation of p53 response	[41]
*Chaer*	Epigenetic regulator in the development of cardiac hypertrophy	[42]
*PINC*	Pregnancy-induced, regulates mammary epithelial differentiation	[43]
*HOTAIR*	Silencing of the *HOXD* locus	[44]
*Braveheart*	Activation of cardiovascular progenitor	[119]
*ANRIL*	Transcriptional silencing to control cellular senescence	[120]
lncRNAs as modulators of proteins and enzyme cofactors		
*NKILA*	NF-κB inactivation by inhibition of IκB phosphorylation	[48]
*lnc-DC*	Regulate genes involved in dendritic cell differentiation	[49]
*ncRNA-a*	Enhancer-like functions on the neighbouring genes	[53]
*CONCR*	Involved in DNA replication and sister-chromatid cohesion	[54]
*Lethe*	Prevents DNA binding of the RelA subunit of NF-κB	[121]
lncRNAs binding DNA/RNA-binding proteins		
*linc-YY1*	YY1-mediated regulation of myogenesis	[64]
*RMST*	SOX2-mediated regulation of pluripotency and neuronal differentiation	[65]
*GAS5*	Regulation of the glucocorticoid response	[58]
*DINO*	Regulation of the DNA-damage-induced p53 response	[59]
*linc-p21*	Repressor in p53-dependent transcriptional response	[23]
*SAMMSON*	Regulation of mitochondrial homeostasis and metabolism	[122]
*LUNAR1*	Enhancing of IGF1 signalling	[123]
lncRNAs forming R-loops and triple helixes		
*TERRA*	Maintenance of the telomeric structure	[72]
*VIM-AS1*	Promotion of transcriptional activation of the *VIM* gene	[75]
*COOLAIR*	Negative regulation of FLC expression and flowering	[77]
*Khps1*	Transcriptional activation of *SPHK1*	[82]
*MEG3*	Transcriptional regulation of genes of the TGF-β pathway	[83]
lncRNAs in higher-order structures		
*Xist*	X chromosome inactivation	[106]
*Firre*	Role in adipogenesis; mediates inter-chromosomal interactions	[107, 124]
*NEAT1*	Nucleation of paraspeckles	[14, 15, 109]
*MALAT1*	Formation of nuclear speckles	[110]

*FLC* flowering locus C, *NF* nuclear factor*, TGF* transforming growth factor, *YY1* Yin Yang 1

However, despite the rapid growth of the field, intriguing questions remain, such as whether all or just a fraction of the existing lncRNAs have a function, or whether this function can be exclusively ascribed to the RNA product of the lncRNA gene. Here, by reviewing the literature, we highlight and discuss different modes of action of lncRNAs in regulating gene expression in the nucleus. We distinguish between mechanisms that are inherent to the RNA molecule or linked to its gene locus, and recapitulate the current evidence that supports the concept that, indeed, the majority of the lncRNAs might be functionally relevant, although highly heterogeneous in their mode of operation.

## Beyond lncRNA transcription: *trans*-regulatory activities of lncRNAs

A significant body of studies where the lncRNA is specifically depleted without perturbing its gene locus supports the notion that many lncRNAs are active species in regulating gene expression of local or distal genes in different organisms, including yeast, plants and higher eukaryotes [6, 20, 21]. Many of these lncRNAs localize to cellular compartments different from their own locus of transcription, including other nuclear domains, the cytoplasm or even polysomes [22]. These observations suggest that some aspects of lncRNA function are strictly dependent on the inherent properties of the RNA molecules, including their ability to fold into different structures and to conduct molecular interactions with other nucleic acids (i.e. RNA and DNA) and proteins. Furthermore, the long sequences of lncRNAs can contain multiple functional domains that interact with different factors coordinating their activity in time and space. For instance, several lncRNAs act in cooperation with heterogeneous nuclear ribonucleoproteins (hnRNPs) [23–26], a large family of RNA-binding proteins involved in different cellular processes, including alternative splicing, mRNA stability, and transcriptional regulation [27]. In addition, it is worth noting the growing evidence showing that some proteins that lack canonical RNA binding domains are able to bind RNA, expanding the number of potential binding partners for lncRNAs, and so extending our view of their regulatory potential over the cell proteome [28]. Here, we discuss some of the most prominent types of functional interactions reported for lncRNAs in the nucleus.

### lncRNA interactions with chromatin complexes

A large repertoire of lncRNAs are able to interact with chromatin-modifying complexes. Given the central role of these complexes during development and disease, this mode of action has been the subject of great attention, and it has been proposed that lncRNAs place these proteins at specific gene loci to achieve appropriate temporal and spatial gene regulation (reviewed in [12, 29, 30]). The lncRNA-containing complexes can promote either selective repression or activation of genes, according to the nature of the chromatin complex [12, 31–33]. For instance, several lncRNAs have been shown to recruit histone H3K4 methyltransferases to promote activation of gene expression [34–36]. Others bind DNA methyltransferases, such as DNMT1 and DNMT3b, and therefore repress transcription by promoting DNA methylation [37–39]. Finally, many lncRNAs have been shown to interact with the polycomb repressive complex 2 (PRC2), which catalyses generation of the H3K27me3 silencing mark [34, 40–45]. For several reasons (reviewed in [46]), PRC2 is the most studied chromatin complex with respect to the functional role of lncRNAs in epigenetic regulation of gene expression. This has largely prompted our knowledge about the biology of the complex, with hundreds of studies published over the past decade, and has also contributed to the debate regarding the direct and specific recruitment of PRC2 by lncRNAs [46, 47], a mechanism that remains to be fully understood. Moreover, it is still unknown whether the lncRNAs that interact with chromatin proteins only act as molecular scaffolds, or whether they also modulate other aspects of the protein functions.

### lncRNAs as modulators of proteins and enzyme cofactors

**Fig. 2:**
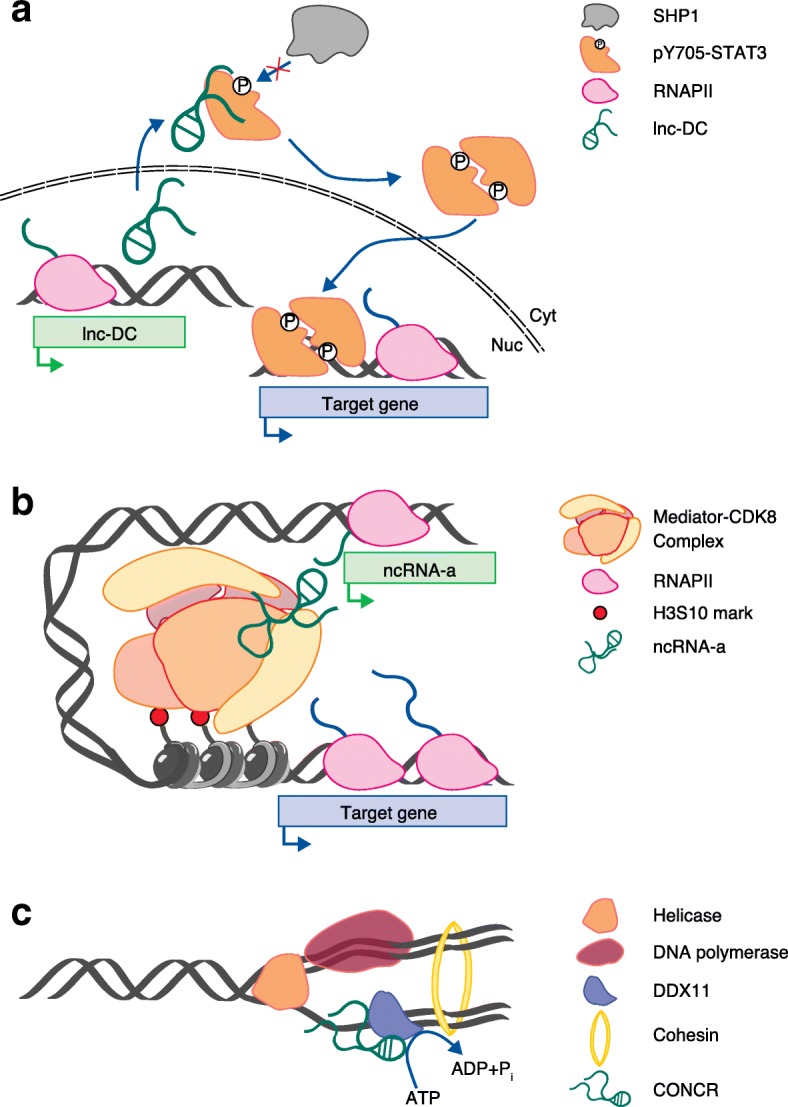
Long noncoding RNAs (lncRNAs) modulate protein activities. **a** The lnc-DC regulates gene expression through a mechanism of action that involves its translocation to the cytoplasm, where it interacts with phosphorylated STAT3 and prevents pY705-STAT3 dephosphorylation by the tyrosine phosphatase SHP1 [49]. **b** The lncRNAs *ncRNA-a3* and *ncRNA-a7* functionally and physically associate with the Mediator complex to promote gene expression of their respective target genes. Either *ncRNA-a* stimulates the kinase activity of the CDK8 subunit of the complex towards the histone H3, catalysing the phosphorylation of the serine 10 (H3S10) [53]. **c** *CONCR* functions in sister-chromatid cohesion by binding and promoting the ATPase activity of DDX11 during DNA replication [54]

In most of the known examples, the detailed mechanisms by which lncRNAs enhance or inhibit the activity of proteins is not completely understood, but are probably diverse and not restricted to the control of protein localization on the chromatin. In some cases, lncRNAs can interfere in the interaction between proteins and protein-modifying enzymes, impeding posttranslational modifications and impacting important signalling pathways. A study describing an NF-kappaB (NF-κB)-interacting lncRNA (*NKILA*) showed that this lncRNA binds NF-κB/IκB in a ternary complex [48]. In this context, using in vitro kinase assays, the addition of *NKILA* to the reaction was found to inhibit IKK-mediated IκB phosphorylation by directly masking the phosphorylation sites, leading to NF-κB inactivation [48]. Also the lncRNA expressed in dendritic cells (*lnc-DC*) regulates protein modification. *lnc-DC* was found to regulate the expression of genes involved in dendritic cell (DC) differentiation [49]. The results suggested a mechanism that involves *linc-DC* interaction with the transcription factor signal transducer and activator of transcription 3 (STAT3) [49]. Such interaction was shown to prevent dephosphorylation of STAT3 at tyrosine Y705 by the tyrosine phosphatase SHP1, and in this way to control the transcriptional programme for differentiation of DCs (Fig. 2a) [49].

Another emerging mode of action of lncRNAs involves their ability to modulate the enzymatic activity of some proteins. Among the early studies identifying and annotating lncRNAs [45, 50, 51], a set of them, named as activating noncoding RNA (ncRNA-a), was found to have enhancer-like functions on the neighbouring protein-coding genes [52]. Two such enhancer-like RNAs (*ncRNA-a3* and *ncRNA-a7*) were found associated with mediator, a transcriptional co-activator complex (Fig. 2b) [53] and increased the occupancy of two different subunits of the complex on the regulated neighbour genes [53]. Interestingly, the lncRNAs were shown to specifically stimulate the kinase activity of mediator towards the histone H3 [53]. The results obtained with *ncRNA-a3* and *ncRNA-a7* [53] suggested that the direct interaction between an lncRNA and a protein is responsible for the proper enzymatic activity of the protein partner.

*CONCR* (cohesion regulator noncoding RNA) is also included in the small number of lncRNAs reported so far to modulate an enzymatic activity [54]. *CONCR* has been shown to physically interact with DEAD/H box protein 11 (DDX11), a DNA helicase involved in DNA replication and sister-chromatid cohesion [55]. *CONCR* and DDX11 colocalize on chromatin, and the silencing of the lncRNA reduces the binding of the helicase at regions of DNA replication [54]. In vitro assays in the presence of purified DDX11 protein and RNA have shown that the binding of *CONCR* to DDX11 promotes the ATPase activity of the helicase [54], suggesting that the lncRNA acts as an RNA effector for the enzyme (Fig. 2c).

Although different in several aspects, such as the heterogenicity of the protein complexes or the final outcome of the interaction, in the situations described above—Mediator–*ncRNA-a* and *CONCR*–DDX11—the lncRNAs have a direct regulatory function on the protein as well as a structural role as the activity of the lncRNA is required for the interaction with the gene loci controlled. As structural changes in proteins caused by cofactor binding or substrate recognition are well known to occur, including in the proteins considered in these examples [56, 57], it can be hypothesized that the functionality of a lncRNA might be ascribed to its ability to function as a cofactor (or effector) of its protein interacting partner. This might also imply a functional conformation in terms of the three-dimensional structure for the lncRNA, although there is a need for stronger evidence to support this interpretation.

### The interplay between lncRNAs and DNA/RNA-binding proteins

**Fig. 3:**
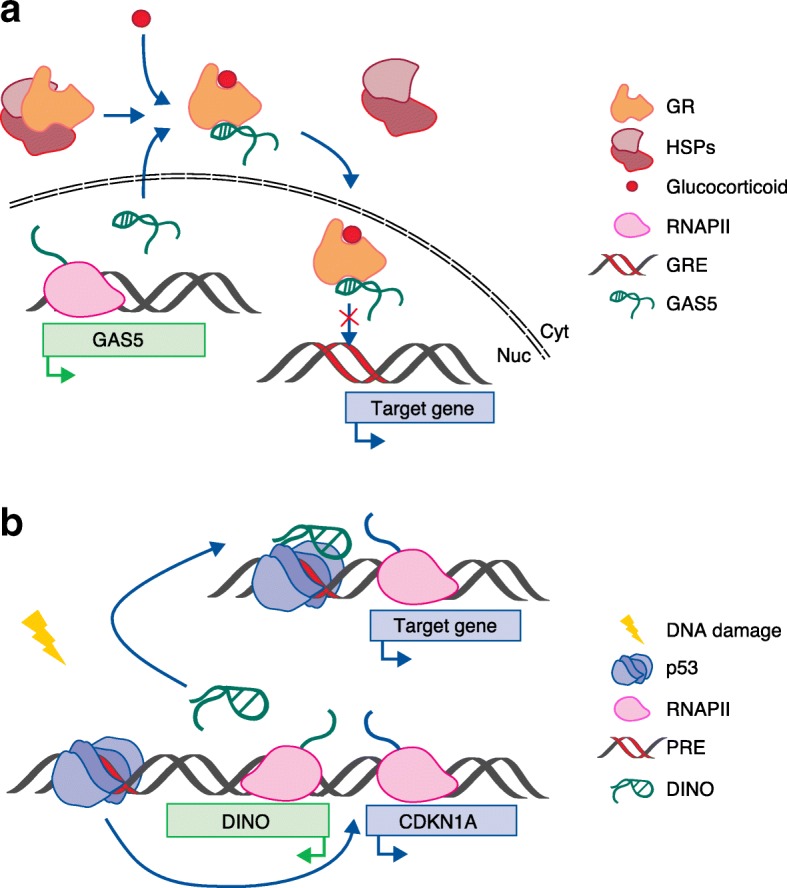
Long noncoding RNAs (lncRNAs) regulate gene expression by interacting with DNA-binding proteins. **a** The lncRNA *GAS5* interacts with the activated glucocorticoid receptor (*GR*), which, following the binding of its ligand and the lncRNA, dissociates from heat-shock proteins (*HSPs*) and translocates to the nucleus. GAS5–GR interaction prevents GR from binding to the glucocorticoid-response elements (*GRE*) contained in responsive genes [58]. **b** DNA-damage-activated tumour suppressor protein p53 induces the transcription of the lncRNA *DINO*, which, in turn, binds and stabilizes p53, promoting the binding of the transcription factor to the p53-response elements (*PRE*) of target genes [59]

Among the proteins that can be modulated by lncRNAs are the transcription factors—the key players of transcriptional regulation. In the most canonical model, gene expression control is thought to be mediated by these DNA-binding proteins (DBPs), whose activation is usually regulated by signalling pathways and whose DNA-binding ability is associated with sequence specificity. By contrast, RNA-binding proteins (RBPs) are generally thought to get involved co- or post-transcriptionally. However, numerous studies have reported a large number of proteins, previously known as DBPs or RBPs, as being equally able to bind both DNA and RNA [28, 58–62]. For such a category of proteins, defined as ‘DNA- and RNA-binding proteins’ (DRBPs), the nature of the interacting RNA could be most diverse, including lncRNA (reviewed in [63]). In this regard, several lncRNAs have been reported to bind previously known DBPs, such as transcription factors, to regulate gene expression. For example, the *linc-YY1*, involved in myogenesis, has been shown to interact with the transcription factor yin yang 1 (YY1) [64], and the lncRNA *RMST* and a panel of other lncRNAs involved in pluripotency maintenance and neuronal differentiation have been shown to interact physically with sex-determining region Y-box 2 (SOX2) [65, 66]. To date, different modes of action have been suggested with respect to the ability of DRBPs to bind DNA and RNA—for example simultaneous or competitive binding. For instance, the lncRNA *GAS5* has been identified as a functional and physical interactor of the glucocorticoid receptor (GR) [58]. *GAS5* was found to interact with the activated GR to suppress its binding to glucocorticoid response elements (GREs) and therefore the expression of the glucocorticoid-responsive genes (Fig. 3a) [58]. Interestingly, the *GAS5*–GR interaction occurs at the DNA-binding domain of the transcription factor, likely through a mimetic GRE that forms in the secondary structure of *GAS5* [58]. This evidence not only suggests that a transcription factor is equally able to bind DNA and RNA, but also indicates that a lncRNA can regulate gene expression by acting as a binding competitor for DBPs.

More recently, while investigating the role of lncRNAs in the p53 pathway, a novel lncRNA, named ‘damage-induced noncoding RNA’ (*DINO*), was identified in the regulation of the DNA-damage-induced p53 response [59]. DINO binds to and stabilizes p53 (Fig. 3b) [59]. Their interaction was found to involve the C-terminus of p53 and to be maintained while p53 is bound to the responsive elements of its target genes [59]. The tumour suppressor p53 is known to have two distinct nucleic acid binding domains, a DNA-binding core domain and a second nucleic acid-binding domain located at the C-terminus, which has long been known to be able to bind RNA, although earlier studies only obtained evidence of binding in vitro [67]. The results obtained for DINO support the notion that p53 can bind simultaneously DNA and RNA and ascribe to the lncRNA a regulatory role in p53-mediated gene expression.

The emerging evidence concerning the active role of lncRNAs on transcription factors suggests that gene expression benefits from an additional mode of regulation. Indeed, the possibility that a lncRNA can bind and regulate a transcription factor, or any other protein involved in gene expression, confers numerous advantages to the cell. For instance, lncRNAs are known to be highly cell and tissue specific [68, 69], which means that, without changing the transcriptional machinery, cell- and tissue-specific regulation of gene expression could be achieved. Alternatively, lncRNA-dependent regulation could also be considered in terms of cost-effectiveness, as RNAs are energetically less expensive to produce for the cell in comparison with proteins. Also, lncRNAs are more rapidly produced than proteins in response to stimuli—a favourable dynamic that could confer faster cellular responses. Moreover, they can act locally at their site of transcription, whereas proteins need to be translated from their encoding RNAs in the cytoplasm and be returned to the nucleus. Although the lncRNA diversity remains underappreciated, and their functionality poorly characterized, the evidence obtained to date and the model proposed suggest that lncRNA–DRBP interactions are as relevant as protein–protein interactions in the regulation of gene expression.

### Direct interaction between lncRNAs and the DNA: R-loops and triple helixes

While the interaction with transcription factors might confer some lncRNAs with the capacity to recognize specific gene loci, the specificity in gene regulation by lncRNAs has also been linked to their ability as nucleic acids to directly bind to the genomic DNA. These interactions might be key for target recognition. One of the mechanisms whereby lncRNA directly binds DNA involves the formation of structures known as R-loops. These are nucleic acid structures that form usually during transcription by reannealing of the nascent RNA to the DNA template, giving rise to a RNA–DNA hybrid and a displaced single-stranded DNA (ssDNA) [70]. When not properly resolved, R-loops might induce DNA damage and genomic instability [71]. For instance, the telomeric lncRNA *TERRA* has been shown to form R-loops at short telomeres in yeast, contributing to the activation of the DNA damage response by promoting recruitment of the Rad51 recombinase in a telomere-length-dependent manner [72]. Interestingly, the murine *TERRA* can also act at distant genomic loci, where it binds and antagonizes the chromatin-remodeler ATRX [73].

In contrast to the above, R-loops can regulate gene expression [74]. For the lncRNAs found to regulate mRNA transcription through the formation of R-loops, the mechanism of regulation is *in cis* as the R-loop formed by the transcription of the lncRNA regulates the expression of the closest protein-coding gene, transcribed in antisense orientation with respect to the lncRNA. This is, for example, the situation observed for the lncRNA *VIM-AS1*, which forms an R-loop around the promoter for the gene encoding vimentin (*VIM*), which, in turn, causes chromatin opening and enhances the binding of transcriptional activators of the NF-κB pathway [75]. By contrast, an R-loop-dependent transcriptional repression has been observed for the *FLOWERING LOCUS C* (*FLC*) in *Arabidopsis thaliana. FLC* expression and flowering are known to be regulated by multiple pathways [76], as well being as negatively regulated by a lncRNA named *COOLAIR* [77]. *COOLAIR* is oriented antisense to the *FLC* gene, and its transcription, and R-loop formation, represses *FLC* expression during prolonged periods of low temperature, preventing in this way the plant from flowering [77]. R-loop stabilization (by binding of the protein NODULIN HOMEOBOX to the ssDNA of the R-loop) in the promoter region of *COOLAIR* has by contrast been found to repress *COOLAIR* transcription, allowing *FLC* expression [77]. To date, our knowledge of the involvement of lncRNAs in R-loop-mediated gene regulation remains very limited. However, considering the high number of sense–antisense paired genes in the genome [78, 79], as well as the ability of some lncRNAs to form R-loops *in trans* [80], it is likely that other lncRNAs will be identified to regulate gene expression via R-loops.

Another way of recognizing chromatin DNA by lncRNAs is the formation of RNA–DNA triplexes [81]. Such structures are generally thought to serve as an anchor for the recruitment of chromatin modifiers in proximity to the gene promoters [81]. This was originally reported for noncoding RNAs produced in the promoter of ribosomal RNAs, which, by forming local triple-helix structures, can recruit the DNA methyltransferase DNMT3b and induce silencing of rRNA genes [38]. Similarly, it has been suggested that RNA–DNA triplexes mediate the recruitment of PRC2 and trithorax-group/mixed-lineage leukemia (TrxG/Mll) protein complexes, both *in cis* (on the *Foxf1* gene) and *in trans* (on the *Pitx2* gene), by the murine lncRNA *Fendrr* [34]. By combining in vitro pull-down and in vivo triplex-capture assays, the lncRNA *Khps1* was shown to form a RNA–DNA triplex [82]. *Khps1* is an antisense RNA of the proto-oncogene *SPHK1* encoding sphingosine kinase 1 and was shown to form the triplex upstream of the transcription start site of *SPHK1*—in this way activating *SPHK1* expression by recruiting the histone acetyltransferase p300/CBP [82]. More recently, other lncRNAs have been reported to recruit chromatin modifiers in an RNA–DNA triplex-dependent manner, at promoters of both neighbouring and distal genes [83–85]. An intriguing possibility is that RNA–DNA triplexes formed by lncRNAs could serve to bypass the need for additional proteins for the specific recruitment of epigenetic factors, although much remains to be understood regarding what drives the lncRNAs to form triplexes and how their formation and resolution dynamics are regulated.

## lncRNA functions linked to their own gene locus

The activity of several noncoding RNAs is tightly connected to their own site of transcription. In this instance, the lncRNA remains in the proximity of its own locus, where its transcription seems to be closely linked to the local association of factors that determine an active or repressive chromatin state. It is thus challenging to uncouple the function of the lncRNA from other inherent attributes of the gene locus, including DNA sequence, transcriptional state and chromatin conformation. Here, we describe mechanisms of lncRNAs in this context.

### Active enhancers as a source of functional ncRNAs

Enhancers are short regions of DNA that are bound by transcription factors and augment the expression of genes contacted through chromatin ‘looping’ [86]. They represent a paradigmatic example of noncoding loci that involves regulation by multiple elements: DNA sequence, three-dimensional (3D) chromatin conformation and RNA transcription. Enhancers generally produce unspliced and non-polyadenylated transcripts named enhancer RNAs (eRNAs). Several studies have evaluated the relationship between enhancer transcripts and gene activation (reviewed in [86]), showing that the levels of eRNAs correlate with mRNA synthesis of the neighbouring genes [51, 87]. Also supporting the notion that eRNAs contribute to enhancer function, eRNA levels correlate with the activity of the enhancer [53, 88, 89], and enhancers that produce eRNAs bind transcriptional co-activators to a higher extent [51, 87], have higher DNase hypersensitivity and marks of active chromatin compared with non-transcribed enhancers [86, 90]. While some work has shown that the eRNA is dispensable for the deposition of active histone marks at enhancers [91], several other studies have provided abundant proof that eRNAs are functional as RNA species. For instance, eRNAs produced from enhancers adjacent to E2-upregulated genes are required for the observed ligand-dependent induction of target coding genes, increasing the strength of specific enhancer–promoter looping [89]. Similar observations have been made for eRNAs transcribed from androgen-receptor-regulated enhancers [92], a gonadotropin hormone α-subunit gene enhancer [93] or *MYOD1* enhancers, where eRNAs regulate the chromatin accessibility of the transcriptional machinery [94]. Although it is still not well understood how eRNAs boost enhancer activity, a study performed in neurons showed that eRNAs might facilitate the transition of paused RNA polymerase II into productive elongation by acting as a decoy for the negative elongation factor (NELF) complex [95]. This evidence suggests that the eRNA can establish local interactions with proteins that either enhance or inhibit the transcriptional activity of the enhancer, constituting an additional element of the enhancer function.

### Cis-regulation by lncRNAs: act of transcription versus RNA product

**Fig. 4:**
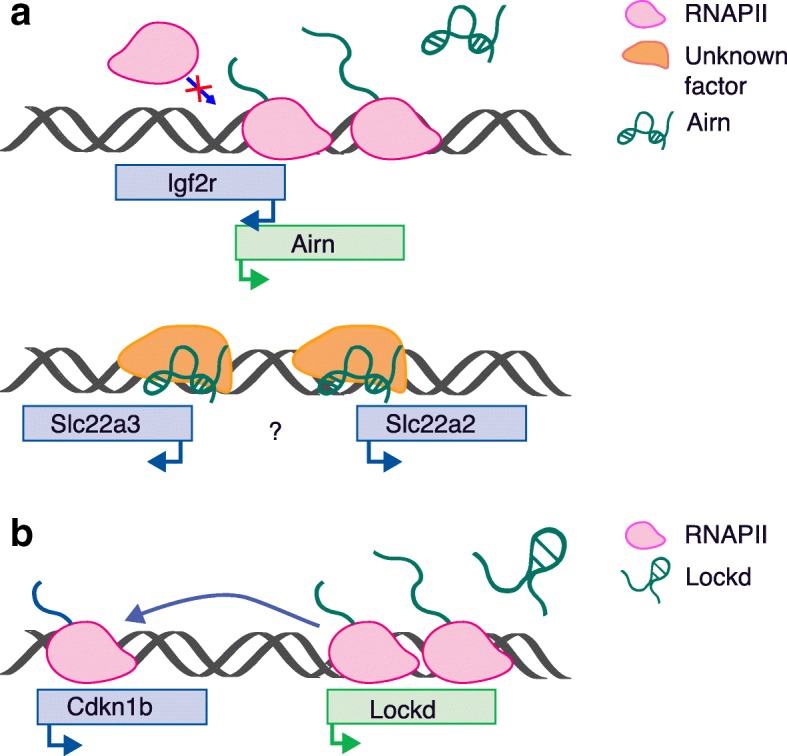
Gene regulation linked to long noncoding RNA (lncRNA) transcription. **a** *Airn* transcription reduces the recruitment of RNAPII to the *Igf2r* promoter, a mechanism known as ‘transcriptional interference’, causing the silencing of the maternal *Igf2r* gene. Transcriptional overlap does not occur at the other imprinted genes in the *Igf2r* cluster—*Slc22a2* and *Slc22a3*—where the silencing properties of *Airn* might reside in its RNA [97, 98]. **b** *Cdkn1b* expression is positively regulated by a *cis*-element of the genomic locus of *Lockd*, whereas the lncRNA is dispensable for this function [99]

A scenario where the function of the noncoding RNA is linked to both the RNA product and the act of transcription could well also apply to some lncRNAs. For example, the lncRNA *Airn*, originally identified in mouse as promoting genomic imprinting of the maternal protein-coding *Igf2r* gene cluster, where *Airn* itself is encoded [96], was later shown to act independently of its RNA [97]. Indeed, by shortening the endogenous *Airn* to different lengths by homologous recombination or repositioning its promoter, *Airn*-mediated silencing of *Igf2r* was shown to be caused by transcriptional interference, where the transcriptional overlap of *Airn* reduced the recruitment of RNAPII to the *Igf2r* promoter, independently of its lncRNA product (Fig. 4a) [97]. However, transcriptional overlap could not explain the imprinting of the other genes in the *Igf2r* cluster—*Slc22a2* and *Slc22a3*—suggesting that at least some of *Airn* silencing properties do reside in its RNA and not just the act of transcription (Fig. 4a) [97, 98].

More recently, using a similar approach to that described for *Airn*, genome editing of the lncRNA *Lockd* locus was used to investigate whether the lncRNA functions through its transcript or DNA elements contained in its genomic locus [99]. The study suggested that *Cdkn1b* is positively regulated by a *cis*-element at the promoter of *Lockd*, whereas the lncRNA is dispensable for this function (Fig. 4b) [99]. The authors of the work could not exclude the possibility that the *Lockd* transcript exerts other functions [99]. However, considering that the transcriptomic analysis, following complete depletion of *Lockd*, showed *Cdkn1b* as the only gene affected significantly, this lncRNA might well represent an actual case of there being a functional byproduct of the *Lockd cis*-element.

Similarly, to gain insight into the relationship between lncRNAs and regulation of gene expression *in cis*, Engreitz and colleagues approached the question on a larger scale, evaluating the effects of the genetic manipulation of 12 lncRNA and six mRNA loci on the expression of nearby genes [100]. By combining CRISPR–Cas9-mediated genome editing (including heterozygous deletions of the promoters, insertion of polyadenylation signals downstream of the transcription start sites, and deletions of exonic, intronic or splicing sites) with a variety of measurements (i.e. RNA-seq, GRO-seq, ChIP-seq), the investigators observed that functional interactions between neighbouring genes are frequent, both for lncRNAs and protein-coding loci, and that such cross-talk relies on different manners of function [100]. These include transcriptional- or co-transcriptional-related processes, such as promoters that act as proximal enhancers, where the RNA has no function per se, or the process of splicing that has *cis*-regulatory functions, partially dependent on the nascent transcript [100]. Although the number of loci evaluated in the study is still very limited considering the thousands of lncRNA–mRNA gene pairs present in the genome [101], none of the lncRNA loci included in the study seemed to require the lncRNA itself for the investigated *cis*-regulatory function [100]. It is interesting to note that similar results were achieved for the protein-coding genes analyzed, which points to the notion that genes, independently of their coding or noncoding status, can exert *cis*-regulatory activities that are independent of the RNA produced. However, it should be considered that, as this study only evaluated the effect of the lncRNAs over their neighbouring genes, no conclusion can be extrapolated regarding the functions of the mature RNA products beyond the regulation of local genes.

## lncRNAs and higher-order structures

The distinction between *cis*- and *trans*-regulatory activity of lncRNAs is not clear when taking into consideration the 3D organization of nuclear compartments. The nuclear conformation can explain co-activation or co-repression of gene loci dependent on the special proximity and the local concentration of the involved factors, including lncRNAs. The genome is in fact a packaged 3D structure that forms higher-order chromatin structures, such as intra- and inter-chromosomal loops and nuclear compartmentalization [102]. This is nowadays known to be the result of specific and regulated interactions between DNA, proteins and RNAs, and lncRNAs are increasingly being recognized as important organizers of this architecture [9, 103, 104]. Moreover, emerging evidence supports the notion that nuclear compartmentalization could be based on liquid-phase separation dependent on the biophysical properties of the molecules that constitute the distinct chromatin domains [105]. In this context, the cooperative association of highly abundant lncRNAs with other RNAs and proteins might be significant for the formation and dynamics of nuclear compartments.

**Fig. 5:**
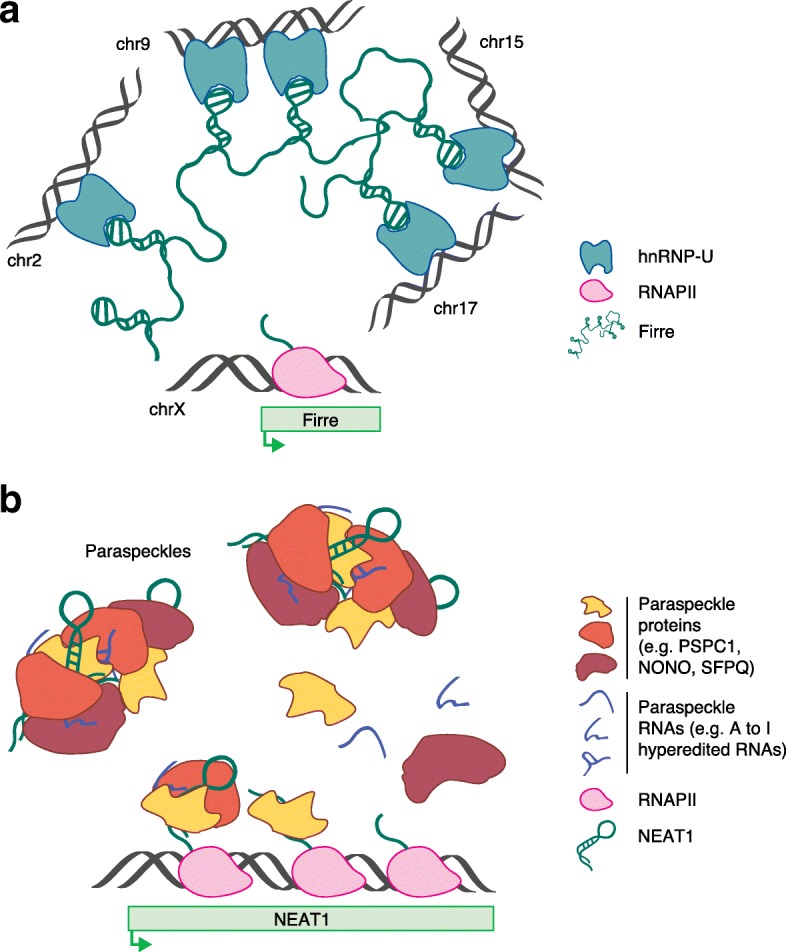
Long noncoding RNAs (lncRNAs) in genome architecture. **a** The lncRNA *Firre* is transcribed from the X chromosome (chrX) and, by binding hnRNP-U, acts as a platform for trans-chromosomal interactions [107]. **b** The lncRNA *NEAT1* functions as an essential structural determinant for the assembly of paraspeckles. The nucleation process begins during the biogenesis of the lncRNA, which acts as a scaffold for the binding of paraspeckle proteins and RNAs, including adenosine to inosine edited RNAs [111, 114]

Among the lncRNAs involved in shaping the 3D structure of the genome, some seem to have a role in the compartmentalization of the DNA in a 3D proximity-guided mechanism, such as *Xist*, which scaffolds a number of proteins that in turn tether the lncRNA to genomic DNA [106], the lncRNA *Firre* that, through the binding of hnRNP-U, acts as a platform for *trans*-chromosomal interactions (Fig. 5a) [107], and the more recently identified trait-relevant (TR)-lincRNAs suggested to regulate proximal TR-protein-coding gene expression by modulating local chromosomal architecture [108]. By contrast, other lncRNAs have been found to form specific nuclear structures, such as the lncRNAs *NEAT1* and *MALAT1* involved in the nucleation of paraspeckles [14, 15, 109] and nuclear speckles [110], respectively.

The role of lncRNAs in nuclear organization is well exemplified by the lncRNA *NEAT1*, which, as mentioned above, drives the formation of nuclear bodies known as paraspeckles. Paraspeckles are subnuclear structures characterized by high local concentrations of specific proteins and RNAs, implicated in the regulation of gene expression by sequestering mRNAs and proteins involved in nuclear processes, including transcription [111, 112]. *NEAT1* is an abundant mono-exonic lncRNA that, following processing at its 3′ end, produces a polyadenylated 3.7-kb *NEAT1_1* isoform and a non-canonically processed 23-kb *NEAT1_2* isoform [111]. Several studies have identified *NEAT1_2* as an essential component for the formation of paraspeckles and have shown that the nucleation process begins during the biogenesis of the lncRNA and that the lncRNA acts as a scaffold for the binding of paraspeckle proteins (Fig. 5b) [14, 15, 109, 113, 114]. Under physiological conditions, the expression of *NEAT1_2*, and thereby paraspeckle formation, occurs in specific cell types involved in pregnancy and lactation [115, 116]. However, the appearance of paraspeckles is known to occur ubiquitously under certain stress conditions [111]. In line with this, *NEAT1* has been identified recently as a direct transcriptional target of p53, and *NEAT1*-dependent formation of paraspeckles has been shown to contribute to the tumour-suppressor function of p53 [117, 118]. In conclusion, as paraspeckle formation requires both the *NEAT1* RNA and its ongoing transcription, and *NEAT1* expression is rapidly regulated in response to stress, the resulting nuclear compartmentalization represents a functional and dynamic manner for controlling gene expression and cellular responses.

## Concluding remarks

Compiling evidence supports the involvement of lncRNAs in the correct execution of gene expression programs, which can be ascribed to three different levels of gene activity: (i) the underlying genomic sequence of the locus, which contains elements able to bind regulatory proteins such as transcription factors; (ii) the act of transcription that can either act as positive feedback or cause transcriptional interference; and (iii) the RNA product itself. The combination of these three dimensions of gene function together with the topological localization in the nucleus is therefore what mediates the effect on gene regulation. However, what makes functional lncRNAs unique is their ability to establish molecular interactions with proteins and nucleic acids to temporally and spatially modulate their activities and/or localization. The examples reviewed here illustrate this mechanistic versatility of lncRNAs—flexibility arising also owing to their evolutionary plasticity. However, only the identification of the RNA sequences and structural elements that confer lncRNAs with these capabilities, as well as the determination of the biochemical and biophysical properties of the lncRNA-containing complexes, will lend further insight into the mechanisms that lncRNAs employ for gene regulation. As our comprehension of lncRNA mechanisms progresses, this will not only expand our view of transcriptional regulation, but also of other important biological processes centred on the chromatin, such as the DNA damage response, DNA repair and DNA replication. Considering the many and diverse functions of lncRNAs, it is therefore not surprising that their alterations contribute to the development and maintenance of many different human diseases. A better understanding of the mechanisms underlying the functions of lncRNAs will help us to understand the pathophysiology of human diseases and to design novel therapeutic strategies and will also benefit fundamental research.

## Abbreviations

CONCR: Cohesion regulator noncoding RNA
DBP: DNA-binding protein
DDX11: DEAD/H box protein 11
DINO: Damage-induced noncoding RNA
DNMT: DNA methyltransferase
DRBP: DNA- and RNA-binding protein
eRNA: Enhancer RNA
FLC: FLOWERING LOCUS C
GRE: Glucocorticoid response element
lncRNA: Long noncoding RNA
MALAT1: Metastasis-associated lung adenocarcinoma transcript 1
ncRNA-a: Activating noncoding RNA
NEAT1: Nuclear enriched abundant transcript 1
NKILA: NF-kappaB-interacting lncRNA
PRC2: Polycomb repressive complex 2
RBP: RNA-binding protein
SOX2: Sex-determining region Y-box 2
STAT3: Signal transducer and activator of transcription 3
YY1: Yin yang 1

## References

[1] Hon CC, Ramilowski JA, Harshbarger J, Bertin N, Rackham OJ, Gough J, Denisenko E, Schmeier S, Poulsen TM, Severin J (2017). An atlas of human long non-coding RNAs with accurate 5' ends. Nature.

[2] Iyer MK, Niknafs YS, Malik R, Singhal U, Sahu A, Hosono Y, Barrette TR, Prensner JR, Evans JR, Zhao S (2015). The landscape of long noncoding RNAs in the human transcriptome. Nat Genet.

[3] Brown CJ, Hendrich BD, Rupert JL, Lafreniere RG, Xing Y, Lawrence J, Willard HF (1992). The human XIST gene: analysis of a 17 kb inactive X-specific RNA that contains conserved repeats and is highly localized within the nucleus. Cell.

[4] Brockdorff N, Ashworth A, Kay GF, McCabe VM, Norris DP, Cooper PJ, Swift S, Rastan S (1992). The product of the mouse Xist gene is a 15 kb inactive X-specific transcript containing no conserved ORF and located in the nucleus. Cell.

[5] Kanduri C (2016). Long noncoding RNAs: Lessons from genomic imprinting. Biochim Biophys Acta.

[6] Fatica A, Bozzoni I (2014). Long non-coding RNAs: new players in cell differentiation and development. Nat Rev Genet.

[7] Fortes P, Morris KV (2016). Long noncoding RNAs in viral infections. Virus Res.

[8] Heo JB, Sung S (2011). Vernalization-mediated epigenetic silencing by a long intronic noncoding RNA. Science.

[9] Engreitz JM, Ollikainen N, Guttman M (2016). Long non–coding RNAs: spatial amplifiers that control nuclear structure and gene expression. Nat Rev Mol Cell Biol.

[10] Rinn JL, Chang HY (2012). Genome regulation by long noncoding RNAs. Annu Rev Biochem.

[11] Bierhoff H, Postepska-Igielska A, Grummt I (2014). Noisy silence: non-coding RNA and heterochromatin formation at repetitive elements. Epigenetics.

[12] Marchese FP, Huarte M (2014). Long non-coding RNAs and chromatin modifiers: their place in the epigenetic code. Epigenetics.

[13] Prasanth KV, Prasanth SG, Xuan Z, Hearn S, Freier SM, Bennett CF, Zhang MQ, Spector DL (2005). Regulating gene expression through RNA nuclear retention. Cell.

[14] Clemson CM, Hutchinson JN, Sara SA, Ensminger AW, Fox AH, Chess A, Lawrence JB (2009). An architectural role for a nuclear noncoding RNA: NEAT1 RNA is essential for the structure of paraspeckles. Mol Cell.

[15] Sunwoo H, Dinger ME, Wilusz JE, Amaral PP, Mattick JS, Spector DL (2009). MEN epsilon/beta nuclear-retained non-coding RNAs are up-regulated upon muscle differentiation and are essential components of paraspeckles. Genome Res.

[16] Yoon JH, Abdelmohsen K, Gorospe M (2013). Posttranscriptional gene regulation by long noncoding RNA. J Mol Biol.

[17] Quinn JJ, Chang HY (2016). Unique features of long non-coding RNA biogenesis and function. Nat Rev Genet.

[18] Wapinski O, Chang HY (2011). Long noncoding RNAs and human disease. Trends Cell Biol.

[19] Huarte M (2015). The emerging role of lncRNAs in cancer. Nat Med.

[20] Bonasio R, Shiekhattar R (2014). Regulation of transcription by long noncoding RNAs. Annu Rev Genet.

[21] Ulitsky I (2016). Evolution to the rescue. using comparative genomics to understand long non-coding RNAs. Nat Rev Genet.

[22] Chen LL (2016). Linking long noncoding RNA localization and function. Trends Biochem Sci.

[23] Huarte M, Guttman M, Feldser D, Garber M, Koziol MJ, Kenzelmann-Broz D, Khalil AM, Zuk O, Amit I, Rabani M (2010). A large intergenic noncoding RNA induced by p53 mediates global gene repression in the p53 response. Cell.

[24] Castellanos-Rubio A, Fernandez-Jimenez N, Kratchmarov R, Luo X, Bhagat G, Green PH, Schneider R, Kiledjian M, Bilbao JR, Ghosh S (2016). A long noncoding RNA associated with susceptibility to celiac disease. Science.

[25] Huang J, Zhang A, Ho TT, Zhang Z, Zhou N, Ding X, Zhang X, Xu M, Mo YY (2016). Linc-RoR promotes c-Myc expression through hnRNP I and AUF1. Nucleic Acids Res.

[26] Carpenter S, Aiello D, Atianand MK, Ricci EP, Gandhi P, Hall LL, Byron M, Monks B, Henry-Bezy M, Lawrence JB (2013). A long noncoding RNA mediates both activation and repression of immune response genes. Science.

[27] Geuens T, Bouhy D, Timmerman V (2016). The hnRNP family: insights into their role in health and disease. Hum Genet.

[28] Castello A, Fischer B, Eichelbaum K, Horos R, Beckmann BM, Strein C, Davey NE, Humphreys DT, Preiss T, Steinmetz LM (2012). Insights into RNA biology from an atlas of mammalian mRNA-binding proteins. Cell.

[29] Rinn JL (2014). lncRNAs: linking RNA to chromatin. Cold Spring Harb Perspect Biol.

[30] Guttman M, Rinn JL (2012). Modular regulatory principles of large non-coding RNAs. Nature.

[31] Morlando M, Ballarino M, Fatica A, Bozzoni I (2014). The role of long noncoding RNAs in the epigenetic control of gene expression. ChemMedChem.

[32] Gendrel AV, Heard E (2014). Noncoding RNAs and epigenetic mechanisms during X-chromosome inactivation. Annu Rev Cell Dev Biol.

[33] Meller VH, Joshi SS, Deshpande N (2015). Modulation of chromatin by noncoding RNA. Annu Rev Genet.

[34] Grote P, Wittler L, Hendrix D, Koch F, Wahrisch S, Beisaw A, Macura K, Blass G, Kellis M, Werber M, Herrmann BG (2013). The tissue-specific lncRNA Fendrr is an essential regulator of heart and body wall development in the mouse. Dev Cell.

[35] Wang KC, Yang YW, Liu B, Sanyal A, Corces-Zimmerman R, Chen Y, Lajoie BR, Protacio A, Flynn RA, Gupta RA (2011). A long noncoding RNA maintains active chromatin to coordinate homeotic gene expression. Nature.

[36] Cabianca DS, Casa V, Bodega B, Xynos A, Ginelli E, Tanaka Y, Gabellini D (2012). A long ncRNA links copy number variation to a polycomb/trithorax epigenetic switch in FSHD muscular dystrophy. Cell.

[37] Mohammad F, Mondal T, Guseva N, Pandey GK, Kanduri C (2010). Kcnq1ot1 noncoding RNA mediates transcriptional gene silencing by interacting with Dnmt1. Development.

[38] Schmitz KM, Mayer C, Postepska A, Grummt I (2010). Interaction of noncoding RNA with the rDNA promoter mediates recruitment of DNMT3b and silencing of rRNA genes. Genes Dev.

[39] Johnsson P, Ackley A, Vidarsdottir L, Lui WO, Corcoran M, Grander D, Morris KV (2013). A pseudogene long-noncoding-RNA network regulates PTEN transcription and translation in human cells. Nat Struct Mol Biol.

[40] Kotzin JJ, Spencer SP, McCright SJ, Kumar DB, Collet MA, Mowel WK, Elliott EN, Uyar A, Makiya MA, Dunagin MC (2016). The long non-coding RNA Morrbid regulates Bim and short-lived myeloid cell lifespan. Nature.

[41] Marin-Bejar O, Marchese FP, Athie A, Sanchez Y, Gonzalez J, Segura V, Huang L, Moreno I, Navarro A, Monzo M (2013). Pint lincRNA connects the p53 pathway with epigenetic silencing by the Polycomb repressive complex 2. Genome Biol.

[42] Wang Z, Zhang XJ, Ji YX, Zhang P, Deng KQ, Gong J, Ren S, Wang X, Chen I, Wang H (2016). The long noncoding RNA Chaer defines an epigenetic checkpoint in cardiac hypertrophy. Nat Med.

[43] Shore AN, Kabotyanski EB, Roarty K, Smith MA, Zhang Y, Creighton CJ, Dinger ME, Rosen JM (2012). Pregnancy-induced noncoding RNA (PINC) associates with polycomb repressive complex 2 and regulates mammary epithelial differentiation. PLoS Genet.

[44] Rinn JL, Kertesz M, Wang JK, Squazzo SL, Xu X, Brugmann SA, Goodnough LH, Helms JA, Farnham PJ, Segal E, Chang HY (2007). Functional demarcation of active and silent chromatin domains in human HOX loci by noncoding RNAs. Cell.

[45] Khalil AM, Guttman M, Huarte M, Garber M, Raj A, Rivea Morales D, Thomas K, Presser A, Bernstein BE, van Oudenaarden A (2009). Many human large intergenic noncoding RNAs associate with chromatin-modifying complexes and affect gene expression. Proc Natl Acad Sci U S A.

[46] Davidovich C, Cech TR (2015). The recruitment of chromatin modifiers by long noncoding RNAs: lessons from PRC2. RNA.

[47] Portoso M, Ragazzini R, Brencic Z, Moiani A, Michaud A, Vassilev I, Wassef M, Servant N, Sargueil B, Margueron R (2017). PRC2 is dispensable for HOTAIR-mediated transcriptional repression. EMBO J.

[48] Liu B, Sun L, Liu Q, Gong C, Yao Y, Lv X, Lin L, Yao H, Su F, Li D (2015). A cytoplasmic NF-kappaB interacting long noncoding RNA blocks IkappaB phosphorylation and suppresses breast cancer metastasis. Cancer Cell.

[49] Wang P, Xue Y, Han Y, Lin L, Wu C, Xu S, Jiang Z, Xu J, Liu Q, Cao X (2014). The STAT3-binding long noncoding RNA lnc-DC controls human dendritic cell differentiation. Science.

[50] Guttman M, Amit I, Garber M, French C, Lin MF, Feldser D, Huarte M, Zuk O, Carey BW, Cassady JP (2009). Chromatin signature reveals over a thousand highly conserved large non-coding RNAs in mammals. Nature.

[51] Kim TK, Hemberg M, Gray JM, Costa AM, Bear DM, Wu J, Harmin DA, Laptewicz M, Barbara-Haley K, Kuersten S (2010). Widespread transcription at neuronal activity-regulated enhancers. Nature.

[52] Orom UA, Derrien T, Beringer M, Gumireddy K, Gardini A, Bussotti G, Lai F, Zytnicki M, Notredame C, Huang Q (2010). Long noncoding RNAs with enhancer-like function in human cells. Cell.

[53] Lai F, Orom UA, Cesaroni M, Beringer M, Taatjes DJ, Blobel GA, Shiekhattar R (2013). Activating RNAs associate with Mediator to enhance chromatin architecture and transcription. Nature.

[54] Marchese FP, Grossi E, Marin-Bejar O, Bharti SK, Raimondi I, Gonzalez J, Martinez-Herrera DJ, Athie A, Amadoz A, Brosh RM, Huarte M (2016). A long noncoding RNA regulates sister chromatid cohesion. Mol Cell.

[55] Bharti SK, Khan I, Banerjee T, Sommers JA, Wu Y, Brosh RM (2014). Molecular functions and cellular roles of the ChlR1 (DDX11) helicase defective in the rare cohesinopathy Warsaw breakage syndrome. Cell Mol Life Sci.

[56] Allen BL, Taatjes DJ (2015). The Mediator complex: a central integrator of transcription. Nat Rev Mol Cell Biol.

[57] Hossain DM, Panda AK, Manna A, Mohanty S, Bhattacharjee P, Bhattacharyya S, Saha T, Chakraborty S, Kar RK, Das T (2013). FoxP3 acts as a cotranscription factor with STAT3 in tumor-induced regulatory T cells. Immunity.

[58] Kino T, Hurt DE, Ichijo T, Nader N, Chrousos GP (2010). Noncoding RNA gas5 is a growth arrest- and starvation-associated repressor of the glucocorticoid receptor. Sci Signal.

[59] Schmitt AM, Garcia JT, Hung T, Flynn RA, Shen Y, Qu K, Payumo AY, Peres-da-Silva A, Broz DK, Baum R (2016). An inducible long noncoding RNA amplifies DNA damage signaling. Nat Genet.

[60] Lanz RB, McKenna NJ, Onate SA, Albrecht U, Wong J, Tsai SY, Tsai MJ, O'Malley BW (1999). A steroid receptor coactivator, SRA, functions as an RNA and is present in an SRC-1 complex. Cell.

[61] Di Ruscio A, Ebralidze AK, Benoukraf T, Amabile G, Goff LA, Terragni J, Figueroa ME, De Figueiredo Pontes LL, Alberich-Jorda M, Zhang P (2013). DNMT1-interacting RNAs block gene-specific DNA methylation. Nature.

[62] Hendrickson GD, Kelley DR, Tenen D, Bernstein B, Rinn JL (2016). Widespread RNA binding by chromatin-associated proteins. Genome Biol.

[63] Hudson WH, Ortlund EA (2014). The structure, function and evolution of proteins that bind DNA and RNA. Nat Rev Mol Cell Biol.

[64] Zhou L, Sun K, Zhao Y, Zhang S, Wang X, Li Y, Lu L, Chen X, Chen F, Bao X (2015). Linc-YY1 promotes myogenic differentiation and muscle regeneration through an interaction with the transcription factor YY1. Nat Commun.

[65] Ng SY, Bogu GK, Soh BS, Stanton LW (2013). The long noncoding RNA RMST interacts with SOX2 to regulate neurogenesis. Mol Cell.

[66] Ng SY, Johnson R, Stanton LW (2012). Human long non-coding RNAs promote pluripotency and neuronal differentiation by association with chromatin modifiers and transcription factors. EMBO J.

[67] Riley KJ, Maher LJ (2007). p53 RNA interactions: new clues in an old mystery. RNA.

[68] Cabili MN, Dunagin MC, McClanahan PD, Biaesch A, Padovan-Merhar O, Regev A, Rinn JL, Raj A (2015). Localization and abundance analysis of human lncRNAs at single-cell and single-molecule resolution. Genome Biol.

[69] Cabili MN, Trapnell C, Goff L, Koziol M, Tazon-Vega B, Regev A, Rinn JL (2011). Integrative annotation of human large intergenic noncoding RNAs reveals global properties and specific subclasses. Genes Dev.

[70] Santos-Pereira JM, Aguilera A (2015). R loops: new modulators of genome dynamics and function. Nat Rev Genet.

[71] Sollier J, Cimprich KA (2015). Breaking bad: R-loops and genome integrity. Trends Cell Biol.

[72] Graf M, Bonetti D, Lockhart A, Serhal K, Kellner V, Maicher A, Jolivet P, Teixeira MT, Luke B (2017). Telomere length determines TERRA and R-Loop regulation through the cell cycle. Cell.

[73] Chu HP, Cifuentes-Rojas C, Kesner B, Aeby E, Lee HG, Wei C, Oh HJ, Boukhali M, Haas W, Lee JT (2017). TERRA RNA antagonizes ATRX and protects telomeres. Cell.

[74] Skourti-Stathaki K, Proudfoot NJ (2014). A double-edged sword: R loops as threats to genome integrity and powerful regulators of gene expression. Genes Dev.

[75] Boque-Sastre R, Soler M, Oliveira-Mateos C, Portela A, Moutinho C, Sayols S, Villanueva A, Esteller M, Guil S (2015). Head-to-head antisense transcription and R-loop formation promotes transcriptional activation. Proc Natl Acad Sci U S A.

[76] Kim DH, Sung S (2012). Environmentally coordinated epigenetic silencing of FLC by protein and long noncoding RNA components. Curr Opin Plant Biol.

[77] Sun Q, Csorba T, Skourti-Stathaki K, Proudfoot NJ, Dean C (2013). R-loop stabilization represses antisense transcription at the Arabidopsis FLC locus. Science.

[78] Yelin R, Dahary D, Sorek R, Levanon EY, Goldstein O, Shoshan A, Diber A, Biton S, Tamir Y, Khosravi R (2003). Widespread occurrence of antisense transcription in the human genome. Nat Biotechnol.

[79] Chen J, Sun M, Kent WJ, Huang X, Xie H, Wang W, Zhou G, Shi RZ, Rowley JD (2004). Over 20% of human transcripts might form sense-antisense pairs. Nucleic Acids Res.

[80] Cloutier SC, Wang S, Ma WK, Al Husini N, Dhoondia Z, Ansari A, Pascuzzi PE, Tran EJ (2016). Regulated formation of lncRNA-DNA hybrids enables faster transcriptional induction and environmental adaptation. Mol Cell.

[81] Li Y, Syed J, Sugiyama H (2016). RNA-DNA triplex formation by long noncoding RNAs. Cell Chem Biol.

[82] Postepska-Igielska A, Giwojna A, Gasri-Plotnitsky L, Schmitt N, Dold A, Ginsberg D, Grummt I (2015). LncRNA Khps1 regulates expression of the proto-oncogene SPHK1 via triplex-mediated changes in chromatin structure. Mol Cell.

[83] Mondal T, Subhash S, Vaid R, Enroth S, Uday S, Reinius B, Mitra S, Mohammed A, James AR, Hoberg E (2015). MEG3 long noncoding RNA regulates the TGF-beta pathway genes through formation of RNA-DNA triplex structures. Nat Commun.

[84] O'Leary VB, Ovsepian SV, Carrascosa LG, Buske FA, Radulovic V, Niyazi M, Moertl S, Trau M, Atkinson MJ, Anastasov N (2015). PARTICLE, a triplex-forming long ncRNA, regulates locus-specific methylation in response to low-dose irradiation. Cell Rep.

[85] Kalwa M, Hanzelmann S, Otto S, Kuo CC, Franzen J, Joussen S, Fernandez-Rebollo E, Rath B, Koch C, Hofmann A (2016). The lncRNA HOTAIR impacts on mesenchymal stem cells via triple helix formation. Nucleic Acids Res.

[86] Li W, Notani D, Rosenfeld MG (2016). Enhancers as non-coding RNA transcription units: recent insights and future perspectives. Nat Rev Genet.

[87] De Santa F, Barozzi I, Mietton F, Ghisletti S, Polletti S, Tusi BK, Muller H, Ragoussis J, Wei CL, Natoli G (2010). A large fraction of extragenic RNA pol II transcription sites overlap enhancers. PLoS Biol.

[88] Melo CA, Drost J, Wijchers PJ, van de Werken H, de Wit E, Oude Vrielink JA, Elkon R, Melo SA, Leveille N, Kalluri R (2013). eRNAs are required for p53-dependent enhancer activity and gene transcription. Mol Cell.

[89] Li W, Notani D, Ma Q, Tanasa B, Nunez E, Chen AY, Merkurjev D, Zhang J, Ohgi K, Song X (2013). Functional roles of enhancer RNAs for oestrogen-dependent transcriptional activation. Nature.

[90] Melgar MF, Collins FS, Sethupathy P (2011). Discovery of active enhancers through bidirectional expression of short transcripts. Genome Biol.

[91] Kaikkonen MU, Spann NJ, Heinz S, Romanoski CE, Allison KA, Stender JD, Chun HB, Tough DF, Prinjha RK, Benner C, Glass CK (2013). Remodeling of the enhancer landscape during macrophage activation is coupled to enhancer transcription. Mol Cell.

[92] Hsieh CL, Fei T, Chen Y, Li T, Gao Y, Wang X, Sun T, Sweeney CJ, Lee GS, Chen S (2014). Enhancer RNAs participate in androgen receptor-driven looping that selectively enhances gene activation. Proc Natl Acad Sci U S A.

[93] Pnueli L, Rudnizky S, Yosefzon Y, Melamed P (2015). RNA transcribed from a distal enhancer is required for activating the chromatin at the promoter of the gonadotropin alpha-subunit gene. Proc Natl Acad Sci U S A.

[94] Mousavi K, Zare H, Dell'orso S, Grontved L, Gutierrez-Cruz G, Derfoul A, Hager GL, Sartorelli V (2013). eRNAs promote transcription by establishing chromatin accessibility at defined genomic loci. Mol Cell.

[95] Schaukowitch K, Joo JY, Liu X, Watts JK, Martinez C, Kim TK (2014). Enhancer RNA facilitates NELF release from immediate early genes. Mol Cell.

[96] Sleutels F, Zwart R, Barlow DP (2002). The non-coding Air RNA is required for silencing autosomal imprinted genes. Nature.

[97] Latos PA, Pauler FM, Koerner MV, Senergin HB, Hudson QJ, Stocsits RR, Allhoff W, Stricker SH, Klement RM, Warczok KE (2012). Airn transcriptional overlap, but not its lncRNA products, induces imprinted Igf2r silencing. Science.

[98] Sleutels F, Tjon G, Ludwig T, Barlow DP (2003). Imprinted silencing of Slc22a2 and Slc22a3 does not need transcriptional overlap between Igf2r and Air. EMBO J.

[99] Paralkar VR, Taborda CC, Huang P, Yao Y, Kossenkov AV, Prasad R, Luan J, Davies JO, Hughes JR, Hardison RC (2016). Unlinking an lncRNA from its associated cis element. Mol Cell.

[100] Engreitz JM, Haines JE, Perez EM, Munson G, Chen J, Kane M, McDonel PE, Guttman M, Lander ES (2016). Local regulation of gene expression by lncRNA promoters, transcription and splicing. Nature.

[101] Sigova AA, Mullen AC, Molinie B, Gupta S, Orlando DA, Guenther MG, Almada AE, Lin C, Sharp PA, Giallourakis CC, Young RA (2013). Divergent transcription of long noncoding RNA/mRNA gene pairs in embryonic stem cells. Proc Natl Acad Sci U S A.

[102] Nunez E, Fu XD, Rosenfeld MG (2009). Nuclear organization in the 3D space of the nucleus--cause or consequence?. Curr Opin Genet Dev.

[103] Cheng L, Ming H, Zhu M, Wen B (2016). Long noncoding RNAs as organizers of nuclear architecture. Sci China Life Sci.

[104] Mele M, Rinn JL (2016). "Cat's Cradling" the 3D Genome by the act of LncRNA transcription. Mol Cell.

[105] Strom AR, Emelyanov AV, Mir M, Fyodorov DV, Darzacq X, Karpen GH (2017). Phase separation drives heterochromatin domain formation. Nature.

[106] Engreitz JM, Pandya-Jones A, McDonel P, Shishkin A, Sirokman K, Surka C, Kadri S, Xing J, Goren A, Lander ES (2013). The Xist lncRNA exploits three-dimensional genome architecture to spread across the X chromosome. Science.

[107] Hacisuleyman E, Goff LA, Trapnell C, Williams A, Henao-Mejia J, Sun L, McClanahan P, Hendrickson DG, Sauvageau M, Kelley DR (2014). Topological organization of multichromosomal regions by the long intergenic noncoding RNA Firre. Nat Struct Mol Biol.

[108] Tan JY, Smith AA, Ferreira Da Silva M, Matthey-Doret C, Rueedi R, Sonmez R, Ding D, Kutalik Z, Bergmann S, Marques AC (2017). Cis-acting complex-trait-associated lincRNA expression correlates with modulation of chromosomal architecture. Cell Rep.

[109] Sasaki YT, Ideue T, Sano M, Mituyama T, Hirose T (2009). MENepsilon/beta noncoding RNAs are essential for structural integrity of nuclear paraspeckles. Proc Natl Acad Sci U S A.

[110] Tripathi V, Ellis JD, Shen Z, Song DY, Pan Q, Watt AT, Freier SM, Bennett CF, Sharma A, Bubulya PA (2010). The nuclear-retained noncoding RNA MALAT1 regulates alternative splicing by modulating SR splicing factor phosphorylation. Mol Cell.

[111] Naganuma T, Hirose T (2013). Paraspeckle formation during the biogenesis of long non-coding RNAs. RNA Biol.

[112] Yamazaki T, Hirose T (2015). The building process of the functional paraspeckle with long non-coding RNAs. Front Biosci (Elite Ed).

[113] Naganuma T, Nakagawa S, Tanigawa A, Sasaki YF, Goshima N, Hirose T (2012). Alternative 3'-end processing of long noncoding RNA initiates construction of nuclear paraspeckles. EMBO J.

[114] Mao YS, Sunwoo H, Zhang B, Spector DL (2011). Direct visualization of the co-transcriptional assembly of a nuclear body by noncoding RNAs. Nat Cell Biol.

[115] Nakagawa S, Shimada M, Yanaka K, Mito M, Arai T, Takahashi E, Fujita Y, Fujimori T, Standaert L, Marine JC, Hirose T (2014). The lncRNA Neat1 is required for corpus luteum formation and the establishment of pregnancy in a subpopulation of mice. Development.

[116] Standaert L, Adriaens C, Radaelli E, Van Keymeulen A, Blanpain C, Hirose T, Nakagawa S, Marine JC (2014). The long noncoding RNA Neat1 is required for mammary gland development and lactation. RNA.

[117] Adriaens C, Standaert L, Barra J, Latil M, Verfaillie A, Kalev P, Boeckx B, Wijnhoven PW, Radaelli E, Vermi W (2016). p53 induces formation of NEAT1 lncRNA-containing paraspeckles that modulate replication stress response and chemosensitivity. Nat Med.

[118] Idogawa M, Ohashi T, Sasaki Y, Nakase H, Tokino T (2017). Long non-coding RNA NEAT1 is a transcriptional target of p53 and modulates p53-induced transactivation and tumor-suppressor function. Int J Cancer.

[119] Klattenhoff CA, Scheuermann JC, Surface LE, Bradley RK, Fields PA, Steinhauser ML, Ding H, Butty VL, Torrey L, Haas S (2013). Braveheart, a long noncoding RNA required for cardiovascular lineage commitment. Cell.

[120] Yap KL, Li S, Munoz-Cabello AM, Raguz S, Zeng L, Mujtaba S, Gil J, Walsh MJ, Zhou MM (2010). Molecular interplay of the noncoding RNA ANRIL and methylated histone H3 lysine 27 by polycomb CBX7 in transcriptional silencing of INK4a. Mol Cell.

[121] Rapicavoli NA, Qu K, Zhang J, Mikhail M, Laberge RM, Chang HY (2013). A mammalian pseudogene lncRNA at the interface of inflammation and anti-inflammatory therapeutics. Elife.

[122] Leucci E, Vendramin R, Spinazzi M, Laurette P, Fiers M, Wouters J, Radaelli E, Eyckerman S, Leonelli C, Vanderheyden K (2016). Melanoma addiction to the long non-coding RNA SAMMSON. Nature.

[123] Trimarchi T, Bilal E, Ntziachristos P, Fabbri G, Dalla-Favera R, Tsirigos A, Aifantis I (2014). Genome-wide mapping and characterization of Notch-regulated long noncoding RNAs in acute leukemia. Cell.

[124] Sun L, Goff LA, Trapnell C, Alexander R, Lo KA, Hacisuleyman E, Sauvageau M, Tazon-Vega B, Kelley DR, Hendrickson DG (2013). Long noncoding RNAs regulate adipogenesis. Proc Natl Acad Sci U S A.

